# High density integration of stretchable inorganic thin film transistors with excellent performance and reliability

**DOI:** 10.1038/s41467-022-32672-8

**Published:** 2022-08-24

**Authors:** Himchan Oh, Ji-Young Oh, Chan Woo Park, Jae-Eun Pi, Jong-Heon Yang, Chi-Sun Hwang

**Affiliations:** ICT Creative Research Laboratory, Electronics and Telecommunications Research Institute (ETRI), Daejeon, 34129 Republic of Korea

**Keywords:** Electronic devices, Semiconductors, Electrical and electronic engineering

## Abstract

Transistors with inorganic semiconductors have superior performance and reliability compared to organic transistors. However, they are unfavorable for building stretchable electronic products due to their brittle nature. Because of this drawback, they have mostly been placed on non-stretchable parts to avoid mechanical strain, burdening the deformable interconnects, which link these rigid parts, with the strain of the entire system. Integration density must therefore be sacrificed when stretchability is the first priority because the portion of stretchable wirings should be raised. In this study, we show high density integration of oxide thin film transistors having excellent performance and reliability by directly embedding the devices into stretchable serpentine strings to defeat such trade-off. The embedded transistors do not hide from deformation and endure strain up to 100% by themselves; thus, integration density can be enhanced without sacrificing the stretchability. We expect that our approach can create more compact stretchable electronics with high-end functionality than before.

## Abstract (web summary)

Transistors with inorganic semiconductors have superior performance than organics. However, they are brittle and thus unfavorable for building deformable electronics. Here, authors directly embed such inorganic thin film transistors into serpentine strings to realize highly stretchable and miniaturized electronic circuits.

## Introduction

Stretchable electronics not only expand their dimensions, but also the innovative possibilities and creative experiences of users. Electronic skins are one representative example of such interesting application^1–3^. They can stretch along with fingers to make robots more like humans by feeling textures and forces. Moreover, conformal heaters can warm the cold robots to human-body temperatures to become more familiar and comfortable to touch^3^.

Transistors are essential building-blocks for such stretchable electronics, as they process various input signals and control the operations of other components^4^. There are two main strategies to make these crucial transistors and circuits stretchable: using intrinsically stretchable materials, including conductors, dielectrics, and semiconductors^5–11^, or placing non-stretchable devices on rigid islands and connecting these islands with stretchable interconnects, which can be made of serpentine shaped bridges, liquid metals, etc.^12–20^. For this, functional islands are almost completely decoupled with strain and stretchable wirings take up almost the entire deformation.

The first strategy, intrinsically stretchable semiconducting materials, has achieved remarkable advances in recent years. They can stretch up to 100% of the strain while exhibiting mobility above 1 cm^2^ V^−1^ s^−1^. Moreover, organic materials that are photo-patternable and also stretchable have been reported recently, enabling optical lithography-based microfabrication^21^. However, their mobility is slightly too low for high-speed applications such as image sensors with high frame rates, mobile application processors, etc.

**Fig. 1:**
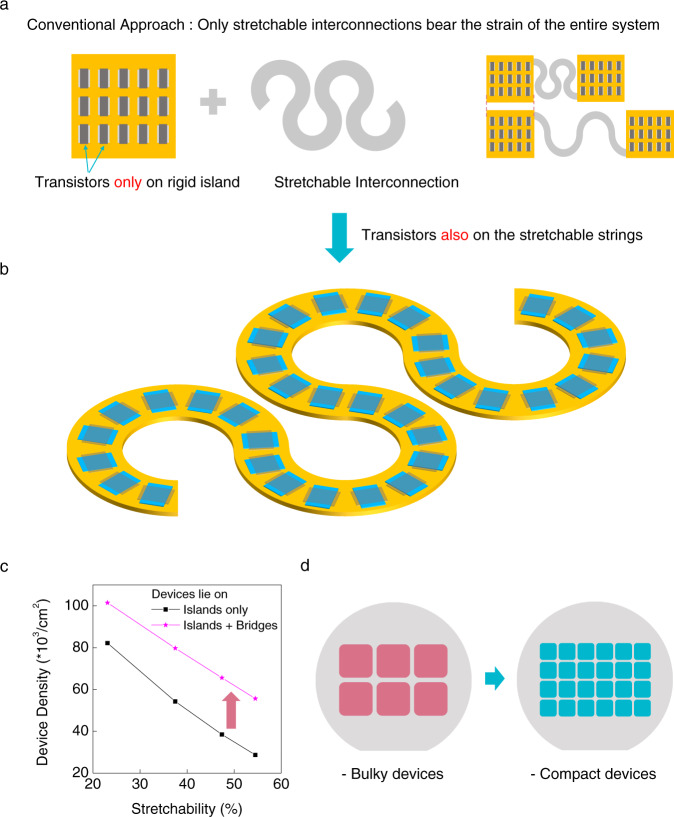
Stretchable inorganic transistors with high integration density. **a** Combination of rigid functional islands and stretchable interconnects to build stretchable electronic system. **b** Devices are arrayed in serpentine strings, where only passive wirings were placed before. **c** Integration density rises when the devices also lie on the serpentine bridges in addition to rigid island (Supplementary Fig. 2 for the details in calculation). **d** Current bulky stretchable electronics can be miniaturized by the proposed approach.

The other strategy has been to focus on high device performance. A combination of rigid islands and stretchable wirings overwhelms the organics in performance since inorganic transistors can be used. Kim et al. reported stretchable complementary metal oxide semiconductor (CMOS) integrated circuits by transfer printing source/drain (SD) doped single crystalline silicon pieces onto polyimide (PI) islands^12,14^. However, the main drawback of this strategy is that the integration density (number of transistors per unit area) is significantly limited because the proportion of stretchable interconnects to rigid islands should be increased to accommodate the high strain on the entire system (Fig. 1a and Supplementary Fig. 1). This leads a bulky stretchable device having many serpentine bridges with few functional islands which is undesirable for both user experience and fabrication cost (Fig. 1d).

Here, we report the large-scale integration of high-performance inorganic thin film transistors (TFTs) by direct fabrication and embedding of devices in serpentine strings to overcome this trade-off between integration density and stretchability (Fig. 1b and Supplementary Fig. 2). We created inorganic TFTs that no longer hide from mechanical strain and robustly endure deformation by themselves. The direct fabrication of TFTs is favorable over transfer printing of separately made devices because it is a far simpler process resulting in better yield and higher throughput. In this context, we developed a simple dual-gate architecture which enables high-performance metal oxide TFTs with excellent reliability for the monolithic integration on the PI supports at low processing temperatures (≤300 °C). These oxide TFTs on serpentine strings can bear actual strain in virtue of PI cladding, which minimizes the stress on the device caused by deformation. We expect our method will realize stretchable large-scale integration (LSI) and enable advanced stretchable devices such as high-fidelity deformable sensors, expandable displays with high resolution, and so on.

## Results and discussion

### Metal oxide TFT with superior performance and stability

Since the first report of In-Ga-Zn-O (IGZO) TFTs, there has been rapid progress in performance and reliability of oxide TFTs. They have begun to be adopted in active matrix displays of portable devices such as smartphones and smartwatches^22–29^.

Stretchable electronics using oxide TFTs also have been steadily reported so far, and we surveyed these studies with emphasis on three main figure of merits, including field effect mobility, stretchability, and device density (Supplementary Table 1)^17,19,30–39^. Among the reports on stretchable oxide TFT arrays, Kim et al. recently reported stretchable a-IGZO TFT with quite high mobility of 24.9 cm^2^ V^−1^ s^−1^, but it can be stretched only up to 30% and just four devices are placed on a substrate size of 25 × 25 mm^2 ^^39^. Meanwhile, Münzenrieder et al. reported highly stretchable a-IGZO TFT array which can be stretched up to 210%, however, the mobility is low as 11.3 cm^2^ V^−1^ s^−1^ and device density is limited to 400 TFTs/cm^2^ which is much lower than 42,000 TFTs/cm^2^ of stretchable organic transistors^17,21^. With such lack of reports on the stretchable TFTs that satisfy aforementioned three indices at the same time, we thus tried to achieve them all in this study.

Though their field effect mobility is high (~20 cm^2^ V^−1^ s^−1^ for IGZO) compared to the organic TFTs, they are still unsatisfactory in applications requiring high processing speed^40,41^. To increase the performance of TFTs, we choose indium tin oxide (ITO) as a high mobility channel material, which is originally known as transparent conducting oxide rather than as a semiconductor. Its higher indium content (In/Sn = 9/1 in weight) than IGZO brings a low effective electron mass because the larger ionic radius of indium (compared to those of zinc and gallium) provides effective percolation pathways for electrons^23,42^. Moreover, the carrier concentration in ITO is higher than IGZO due to the absence of carrier suppressors like gallium, and it also helps the percolative conduction by defeating the potential barriers.

**Fig. 2:**
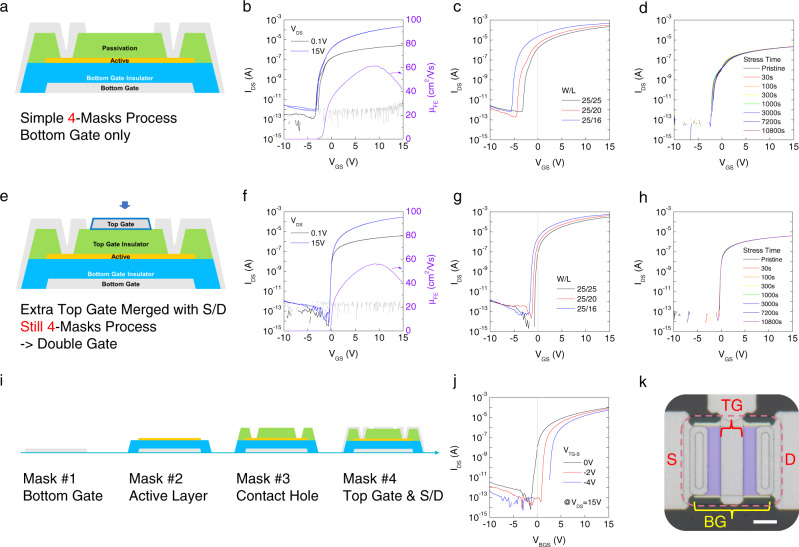
Performance enhanced oxide TFTs. **a** Schematic of bottom gate type oxide TFT. **b** Transfer characteristic and field effect mobility of the bottom gated TFT. **c** Transfer curves of bottom gate TFT with different channel lengths. **d** Sampled transfer curves during the positive gate bias stress test for 3 h. **e** Illustration of simple dual-gate architecture. **f** Transfer characteristic and field effect mobility of dual gated TFT. **g** Transfer curves of dual-gate TFT with various channel lengths. **h** Samplings of transfer characteristics during the positive gate bias stress test for 3 h. **i** Schematics of the fabrication process of oxide TFT along with masks for photo-lithography. **j** Threshold voltage control by separately applying top and bottom gate bias. **k** Optical microscope image of dual gated oxide TFT. Scale bar, 10 μm.

The high electron density in ITO, however, makes TFTs difficult to turn off, even with a high negative gate bias, and also causes a negative shift of the threshold voltage. To achieve proper on/off operation, we control the carrier concentration in ITO by introducing oxygen with argon during the sputtering process (Supplementary Fig. 5). Oxygen vacancies are well-known electron donors in oxide semiconductors along with substitutional dopants like tin in ITO thin films^42^. Therefore, as the content of oxygen vacancies in the oxide semiconductors rises, the electron concentration also increases. We thus introduce oxygen gas during the sputtering of ITO to reduce the formation of oxygen vacancies in the deposited film. In addition to this, its physical thickness is kept under 6 nm to fully deplete electrons. If an ITO film gets thicker, it becomes harder to deplete electrons in it by the gate bias. In other words, electrons can only be partially depleted and the back channel (opposite side of the gate electrode) remains not depleted. This can cause high off current and negative shift of threshold voltage. To overcome this, Li et al. reported ultra-thin (down to 4 nm) ITO channel for high-performance TFTs with proper on/off operations^42^. We used this approach to first fabricate ITO TFTs with a bottom gate structure including a passivation layer on top of the ITO which also act as an etch stopper (Fig. 2a). This shows a remarkable field effect mobility of over 60 cm^2^ V^−1^ s^−1^ with a 100-nm-thick SiO_2_ gate insulator, which is far higher than that of IGZO (Fig. 2b). However, the threshold voltage was quite negative (−4 V), as earlier concerned, and became severer, exceeding −5 V, as the channel length shortened from 25 to 16 μm. Oxide semiconductors which are covered by gate dielectrics or etch stoppers like in our devices can be doped during the dry etching of such insulators to open the hole for SD contacts^43^. This is because the exposed parts of oxide semiconductors are damaged by the plasma, and donor defects like oxygen vacancies are thus formed at there. The increased electrons can diffuse from these doped regions (SD sides) toward the center of the channel. This leads to the rise of carrier concentration in the channel and thus to negative shift of threshold voltage^44,45^. The impact of carrier diffusion from SD regions becomes severer as the channel length gets shorter (Supplementary Fig. 6) because the portion of areas where the electrons are diffused to rises compared to the intrinsic part. This issue is unfavorable for the scaling down of TFTs, which is essential for high-density integration. We also tested the electrical stability of this device by applying positive gate bias stress for 3 h. while being quite stable, there was an obvious positive shift of threshold voltage due to electron trapping at the interface between the semiconductor and gate dielectric^46,47^. Such positive bias instability worsens as the thickness of the semiconductor decreases, such as our 6-nm-thick ITO channel, because the charge density rises within a thinner active layer at a given gate bias. Band bending also becomes steeper than in a thicker channel^48–50^.

An additional gate is one of the most powerful solutions for the above-mentioned problems: the threshold voltage shift with channel length scaling and bias stress-induced instability. An extra gate solves these issues by enhancing the gate controllability on the channel and inducing soft band bending within the semiconductor while bias stress is applied^51–53^. The second gate, however, requires extra steps, including thin film deposition, photo-lithography, and patterning which increases the cost and lowers the throughput.

To avoid additional fabrication steps for the second gate, we merged the extra top gate with SD by creating a space between them, as depicted in Fig. 2e; thus, the number of masks is kept at four, as in the single gate process. The idea is simple, but its impact on the device performance and reliability is dramatic. There is no hysteresis at all in the transfer characteristic of this double gate TFT, as shown in Fig. 2f, unlike the bottom gated one, and the threshold voltages also become close to zero, even at the shortest channel length of 16 μm, thanks to the enhanced gate controllability (Fig. 2g). Moreover, it can endure three hours of a bias stress test without any threshold voltage shift due to softer band bending in the channel by the gates on both sides. Additionally, the threshold voltage can be controlled by applying bias separately on the top and bottom gate, which is useful for circuit configuration (Fig. 2j).

As mentioned before, there is a space between the extra top gate and SD in order to merge them into a single mask. Making this gap as small as possible is the simplest way to get the highest device performance because the portion of channel turned-on by the top gate is maximized. We thus set this space to 3 μm, which is well-guaranteed minimum feature size of our lithography tools. Meanwhile, we also found that the field-effect mobility was still high (48.6 cm^2^ V^−1^ s^−1^) compared to IGZO TFTs when this gap is even doubled to 6 μm. Threshold voltages also did not change at all regardless of the space between top gate and SD (Supplementary Fig. 7). In addition to this, we can adjust the overlap between the bottom gate and the SD to reduce the parasitic capacitance between them for high-frequency operations. We tested the TFTs with various overlap lengths (7, 2, and 0 μm) to study the impact of this parameter on the device characteristics (Supplementary Fig. 8). For the non-overlapped one, the field effect mobility decreases to 45.9 cm^2^ V^−1^ s^−1^ because the electrons in the part of ITO where the contacts are made with SD metals, cannot be accumulated in high density due to the absence of the gate field. Although there is a loss in field effect mobility for this case, it can be compensated by the reduction in parasitic capacitance in the aspect of circuit operation.

### Direct embedding of oxide TFTs into serpentine strings

**Fig. 3:**
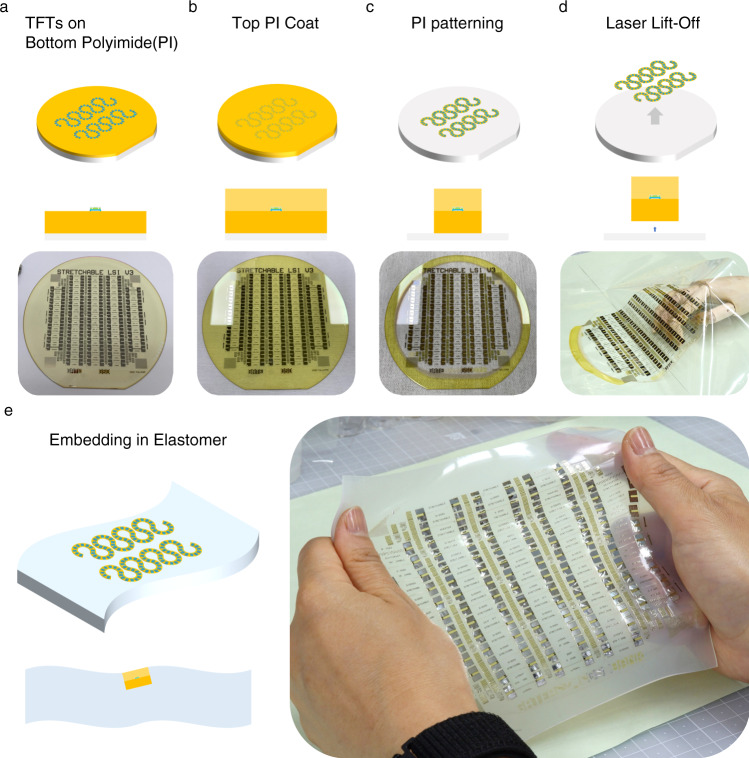
6-inch wafer scale fabrication of integrated inorganic TFT arrays and whole transfer thereof to elastomer. **a** TFTs arrayed in serpentine form on the PI substrate. **b** Top PI coating over the TFTs with the same thickness as the bottom PI to place devices in a neutral plane. **c** Etch out of two PI coatings in serpentine form. **d**, **e** Detachment of PI cladded devices by the LLO technique and transfer thereof to elastomer.

Figure 3 summarizes the process to embed TFTs into serpentine bridges, incorporating steps for PI cladding, laser lift-off (LLO), and transfer to elastomer. A 6-inch glass wafer is used as a substrate and is covered with 2.5 μm-thick PI film by spin coating which serve as the bottom part of cladding. PI is an ideal choice for flexible or deformable electronics thanks to its mechanical durability and substantial thermal resistance.

**Fig. 4:**
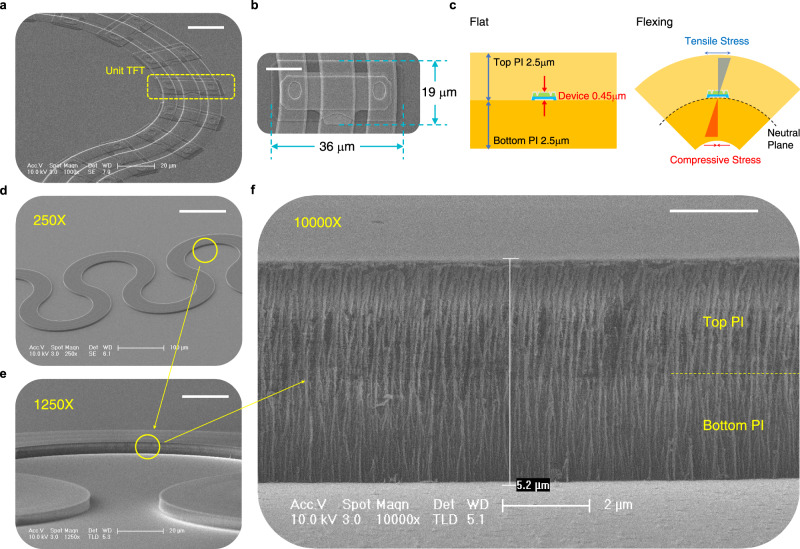
SEM images of TFTs and PI serpentine strings. **a** Oxide TFTs arrayed in serpentine form before the top PI coating. Scale bar, 20 μm. **b** A unit TFT in the serpentine array. Scale bar, 10 μm. **c** Schematics of a PI cladded oxide TFT when in flat and flexed situations. **d**, **e** PI serpentine strings after the etch of top and bottom PIs. Scale bars, 100 and 20 μm for **d**, **e**, respectively. **f** Sidewall of PI serpentine bridges and boundary between top and bottom PIs. Scale bar, 2 μm.

We designed the unit device to be 36 × 19 μm^2^ in size, including pads for the SD and gate metals, and the channel length and width of were set to be 20 and 5 μm, respectively (Supplementary Figs. 9 and 10). An array was then made with the device in serpentine form (Fig. 4a, b). After fabrication of the TFT arrays, they were coated with a second PI with the same thickness of 2.5 μm to place the devices near the center of the 5 μm-thick PI cladding (Fig. 4c). This middle part of the PI cladding between the top and bottom PIs becomes the neutral plane where the internal stress goes to zero when the PI cladding is flexed^54,55^. The TFTs near the neutral plane of cladding, therefore, experience much less stress when the serpentine strings are stretched and twisted^55^. We also performed finite element analysis (FEA) to study the impact of PI cladding on the deformation, and confirmed that the top PI significantly reduces the stress on the TFT array when it is stretched. (Supplementary Fig. 11). A far thinner device thickness (≤0.45 μm) than the PI cladding (5 μm) helps the entire body of the TFTs to not significantly depart from the neutral plane. Interestingly, even the structure of our TFTs are symmetric in the vertical direction, i.e., the channel is sandwiched with two gates and dielectrics (Fig. 2e); thus, it also helps that the semiconductor and the two interfaces with dielectrics, can lie near the neutral plane. After the second PI coat, polymer cladding is completed by patterning the whole PI by an oxygen plasma etch in serpentine shape (Fig. 4d, e) and the boundary between the top and bottom polymer can be found (Fig. 4f).

The PI film was then transferred from 6-inch glass wafer to an elastomer. The LLO technique, commonly used in the fabrication of flexible electronic products, was used to separate our device from the glass substrate. The device was then transferred onto Ecoflex elastomer, a widely used elastomer in stretchable electronic studies. Figure 3d, e show the process of successfully transferring whole devices from a 6-inch wafer to an elastomer on a large scale.

**Fig. 5:**
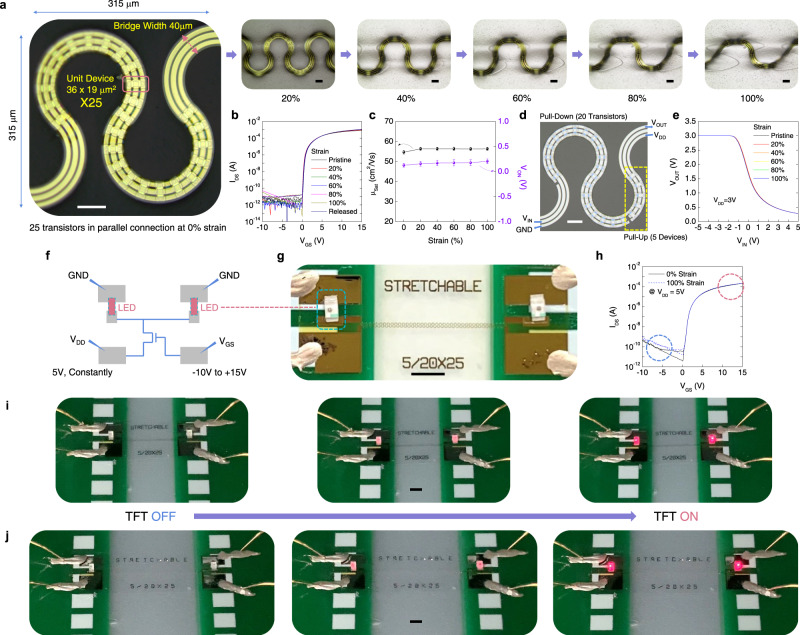
Stretchability of oxide TFTs in serpentine strings. **a** Optical microscope images of 25 unit-devices in serpentine string stretched up to 100% strain. Scale bars, 50 μm. **b** Transfer characteristics of 25 transistors in parallel connection upon the strain from 0 to 100%. **c** Field effect mobility and turn-on voltage of TFTs during the stretching test. Error bar represents standard deviation. **d**, **e** Optical microscope image of stretchable inverter and its voltage transfer curves under strain up to 100%. Scale bar, 50 μm. **f**, **g** Schematic and photograph of two LED pixels driven by stretchable oxide TFTs, embedded in the serpentine bridge. Scale bar, 2 mm. **h** Current–voltage curves of LED-connected TFTs measured before and after the stretching. **i**, **j** Red lights from two LED pixels as the gate voltages swept to the positive side at 0% and 100% strains. Scale bars, 2 mm for both (**i**, **j**).

For lithography, a traditional mask aligner was used with photomasks having 3 μm minimum feature size (line and space). Submicron feature is capable with this aligner if masks strongly contact with substrates. However, this often leads to mask contamination and lowers the yield in large-scale fabrication. We thus used proximity mode to avoid mask/wafer contact for the prevention of mask contamination. The resolution in this mode is then limited to about 2.5 μm. Even with such low-resolution technique, we could integrate more than 30 unit-devices in a 315 × 315 μm^2^ sized square (>30,000 TFTs/cm^2^) where one and half periods of serpentine string (40 μm-wide) can be occupied (Fig. 5a). This is a far higher integration density than the aforementioned stretchable array with Si transistors which are only placed on the rigid islands (<2000 Si transistors/cm^2^)^12^.

The integration density of TFTs on serpentine strings can readily be raised by scaling down the device using lithography tools with higher resolutions, such as i-line steppers or ArF scanners, which are commonly used in semiconductor fabrications, guaranteeing 800 nm or 100 nm resolutions, respectively. Because the process for oxide TFTs is highly compatible with typical semiconductor processes, adoptions of such semiconductor lithography tools are also common^40,41^. An IGZO TFT with a 180 nm channel length by a KrF scanner (Slightly lower resolution than ArF scanners) is a representative example at this point^56^. The size of the unit device (36 μm * 19 μm = 684 μm^2^) in our report can be scaled down with an i-line stepper or ArF scanner to 50 μm^2^ or 0.8 μm^2^, respectively.

### Stretchability of integrated TFTs and demonstrations

For the basic mechanical test, we arrayed 25 unit TFTs in parallel connection within the 40-μm-wide serpentine bridges, and stretched it from 0 to 100% strain in 20% intervals (Fig. 5a). Transfer characteristics measured at each interval almost overlap and its field effect mobility and threshold voltage are nearly unchanged after 100% strain (Fig. 5b, c). A cyclic stretching test was also carried out with the harsh conditions of a 100% strain, 1 mm/s strain rate and 10,000 times of deformation. The TFTs cladded with PI successfully handled this repeated stretch and release test (Supplementary Fig. 12 and Video 1).

After confirming such excellent mechanical reliability of TFTs, we built two examples to demonstrate their application in stretchable integrated circuits and displays. Figure 5d shows that the inverter consists of 25 unit TFTs. Among the devices, gates of 5 TFTs were tied with a power supply line (diode connection). The fabricated inverter operated well, even at the 100% strain, and its voltage transfer curves were also nearly unchanged upon deformation (Fig. 5e).

Figure 5f depicts the diagram of two light emitting diodes (LEDs) connected with stretchable TFTs for the display application demonstration. The LED is 1.6 × 0.8 mm^2^ in size and placed at the both sides of stretchable TFTs (Fig. 5g). The driving voltage (*V*_DD_) is supplied through the TFTs, not directly to the LEDs as the ones in the display pixels. Though 5 V is continuously applied to the drain terminal, a negative gate bias completely turns off the TFTs and blocks the current flow to the LEDs (Fig. 5h). As the gate voltage swept to the positive side, the TFTs were turned on and the LED started to shine (Supplementary Video 2 and 3). The brightness of the LED gradually increased as the gate became more positively biased (Fig. 5i, j). The superior stability under deformation was confirmed once more with the overlapping of two current–voltage curves of LED-connected TFTs measured at 0 and 100% strain (Fig. 5h). Each letter of ‘STRETCHABLE’ on the sample is uniformly separated along with the stretching direction. This means that the metal electrodes and TFTs on serpentine strings were also uniformly stretched during the mechanical test.

In summary, we introduced ‘actually’ stretchable inorganic transistors with high performance and excellent electrical and mechanical stability. High integration density (>30,000 Transistors/cm^2^) is achieved by the direct embedding of oxide TFTs into serpentine strings, where passive electrodes were usually placed in former inorganic stretchable arrays. The electrical characteristics were preserved even after stretchable TFTs were stretched to 100% strain, thanks to the PI cladding. In addition, our approach is based on the standard semiconductor/display fabrication techniques. Thus, high yield and uniform device characteristics can be achieved. We expect that our approach paves the way for fabricating highly miniaturized stretchable products requiring high performance and reliability.

## Methods

### Fabrication of PI cladded oxide TFT array

Polyimide varnish (KPI-1500, Komec) was spin-coated on a 6-inch glass wafer at 2500 RPM for 2 min. The wafer was dried at 80 °C for 10 min and baked at 450 °C for an hour with N_2_ purging. As buffer layers, SiN_x_ and SiO_2_ were sequentially deposited by plasma enhanced chemical vapor deposition (PECVD) at 300 °C with a thickness of 10 nm for each. Mo (15 nm) and ITO (5 nm) layers were also sequentially sputtered, and they were patterned as a bottom gate through photo-lithography (MA6, SUSS MicroTec) and the wet etching technique (MA-SO2, Dongwoo Finechem).

This bottom gate was covered with 100-nm-thick PECVD SiO_2_ as gate dielectric at 300 °C and the ITO channel layer was sputtered on the SiO_2_. For ITO deposition, direct-current (DC) plasma power was kept at 150 W and O_2_ was introduced at the flow rate of 1.0 SCCM in addition to Ar as sputtering gas (24 SCCM), to control the carrier concentration in ITO. A 10-nm-thick SiO_2_ was also deposited on the ITO channel to protect the channel layer from damages caused by wet chemicals and plasma during the subsequent etching process. The active layer, consisting of ITO and SiO_2_, patterned by the dry etching technique using a Cl_2_ and Ar gas mixture (50/50 SCCM) and the working pressure and radio-frequency (RF) plasma power were kept at 5 mTorr and 350 W, respectively.

The second gate insulator was then deposited on the active layer using PECVD at 300 °C with a thickness of 140 nm. The contact holes were etched by dry etching using CF_4_ and Ar gases (80/20 SCCM), with a working pressure of 5 mTorr and an RF plasma power of 300 W. The insulating layers, including the top/bottom gate dielectrics and buffers, were etched out at this stage, except for the active area with some margins (as shown by the dashed line in Fig. 2k) to expose the bare polyimide surface. After the etching of dielectrics, Mo (20 nm)/Al (100 nm)/Mo (30 nm) metal layers were sputtered and patterned as SD contacts, extra top gate and measurement pads by wet etching with the same etchant for the bottom gate patterning. Thermal annealing was then carried out for 2 h at 300 °C in a vacuum. Another polyimide varnish (VTEC^TM^ PI-1388, RBI) was spin-coated at 4000 RPM for 3 min and dried at 120 °C for 3 min. The final bake was done at 250 °C for an hour in a vacuum (Fig. 3b). The 10-nm-thick ITO hard mask was sputtered on the top PI and patterned by dry etching with the same conditions for etching of the active layer. Finally, the bottom and top PI were etched through this ITO hard mask in a serpentine shape by O_2_ plasma and an ITO mask was stripped by the same wet etchant for gate patterning (Fig. 3c).

### Transfer of PI cladded oxide TFT array to elastomer

First, the fabricated devices on the glass wafer were laminated with pick-up film (SPV-P-367K, Nitto Denko). They were then detached from the glass wafer by LLO (KORONA^TM^, AP systems) technique. The TFT arrays were thus temporarily transferred to the pick-up film at this step. The elastomer precursor (Ecoflex 00-30, Smooth-On) was poured onto the pick-up film to cover the whole devices on the film, and cured for 3 h at room temperature. After the curing of elastomer, transfer process was completed by detaching the pick-up film (Supplementary Fig. 14).

### Electrical characterization of oxide TFT

Transfer characteristics of TFTs and stress tests were performed in air using a semiconductor device analyzer (B1500A, Keysight). The field-effect mobility in a saturation regime was estimated from the transfer characteristics using the following equation: *I*_D,sat_ = *μ*_FE_(*WC*_i_/2*L*)(*V*_GS_−*V*_th_)^2^ where *I*_D,sat_, *W*, *L*, *C*_i_, *V*_GS_, and *V*_th_ are the drain current in saturation regime, the channel width, the channel length, the gate capacitance per unit area, the gate to source voltage and the threshold voltage, respectively. For single gate TFTs, the *C*_i_ is solely from the 100-nm-thick SiO_2_ bottom gate dielectric. On the other hand, there are two capacitors in dual-gate TFTs; thus, *C*_i_ in this case is the sum of capacitances from the top gate (*C*_TG,i_) and bottom gate (*C*_BG,i_). The field effect mobility of dual-gate TFTs in the present study is somewhat underestimated because the size of the top gate was set equal to the bottom gate for mobility extraction, although the area of the top gate is smaller than that of the bottom gate in the proposed TFT structure due to the offset region. We considered this a stricter and more conservative way to evaluate our TFT because the top gate, including the offset region, actually occupies the same space as the bottom gate and is a fairer comparison to conventional dual-gate TFTs, which have an equivalent size of top and bottom gates.

## Supplementary information

Supplementary Information

Description of Additional Supplementary Files

Supplementary Video 1

Supplementary Video 2

Supplementary Video 3

## Data Availability

The authors declare that all data supporting the findings of this study are available within the article and its Supplementary Information files or from the corresponding author upon reasonable request.
