# Supplementary material for: High density integration of stretchable inorganic thin film transistors with excellent performance and reliability
Source: Nat Commun. 2022 Aug 24;13:4963. doi: 10.1038/s41467-022-32672-8 (PMC9402572; doi:10.1038/s41467-022-32672-8)
Supplement: Supplementary file 1 — Supplementary Information [file 41467_2022_32672_MOESM1_ESM.pdf]

Supplementary Information

High Density Integration of Stretchable Inorganic Thin Film Transistors with Excellent Performance and Reliability

Himchan Oh[1], Ji-Young Oh[1], Chan Woo Park[1], Jae-Eun Pi[1], Jong-Heon Yang[1], and Chi-Sun Hwang[1*]

[1]ICT Creative Research Laboratory, Electronics & Telecommunications Research Institute (ETRI), Daejon 34129, Republic of Korea

[*]Corresponding author, E-mail address : hwang-cs@etri.re.kr

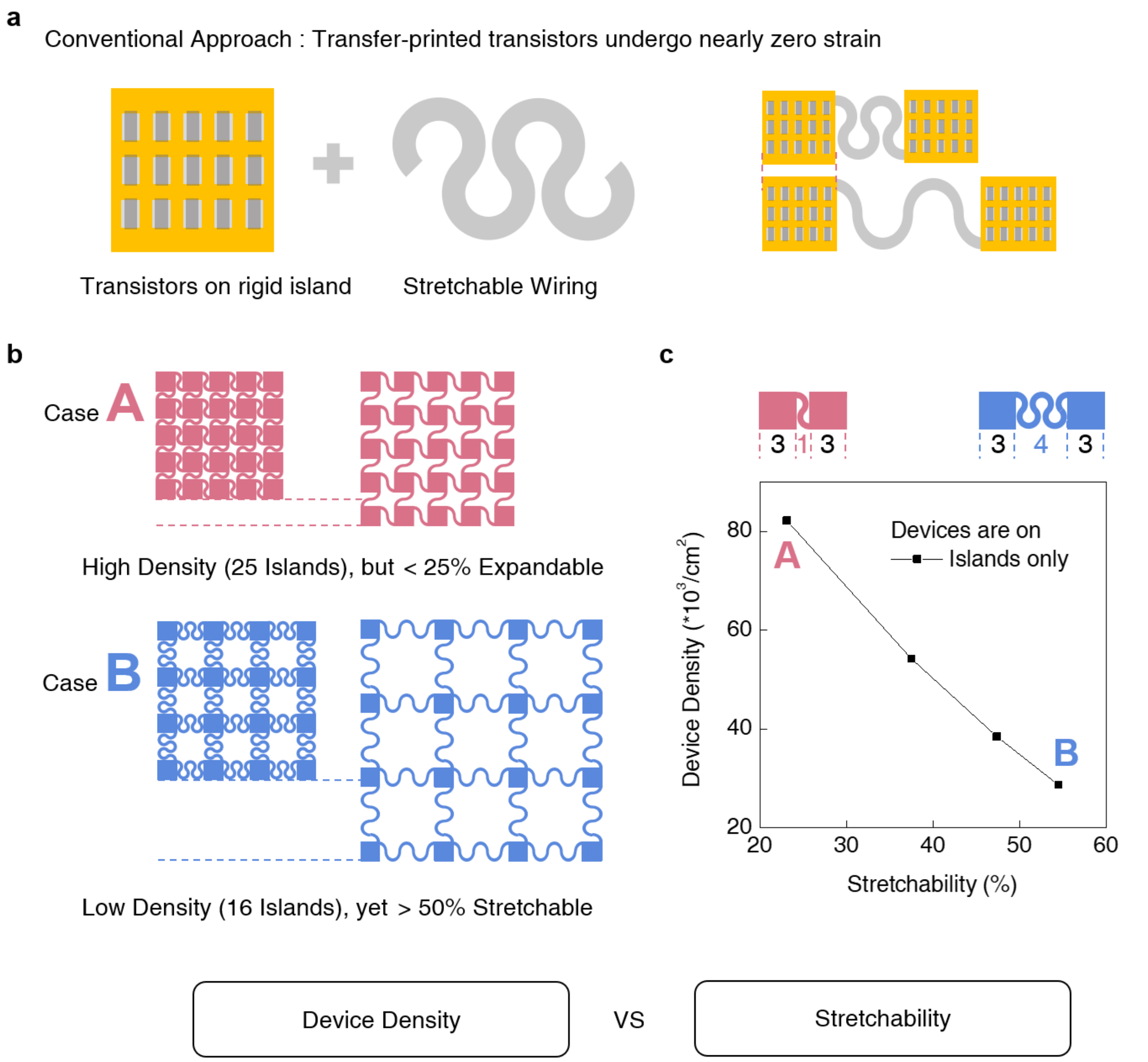

Supplementary Figure 1 | Conventional stretchable integrated electronics and their limitation. a, Combination of rigid functional islands and stretchable interconnects to build stretchable electronic systems. Devices on the islands are nearly impervious to strain while entire systems are elongated. **b**, Trade-off between stretchability and integration density in the conventional approach. Many devices can be integrated for case A than B, because number of islands (25) is higher than that of case B (16). On the other hand, case B can be stretched to over 50 %, whereas case A can endure less than 25 % strain.

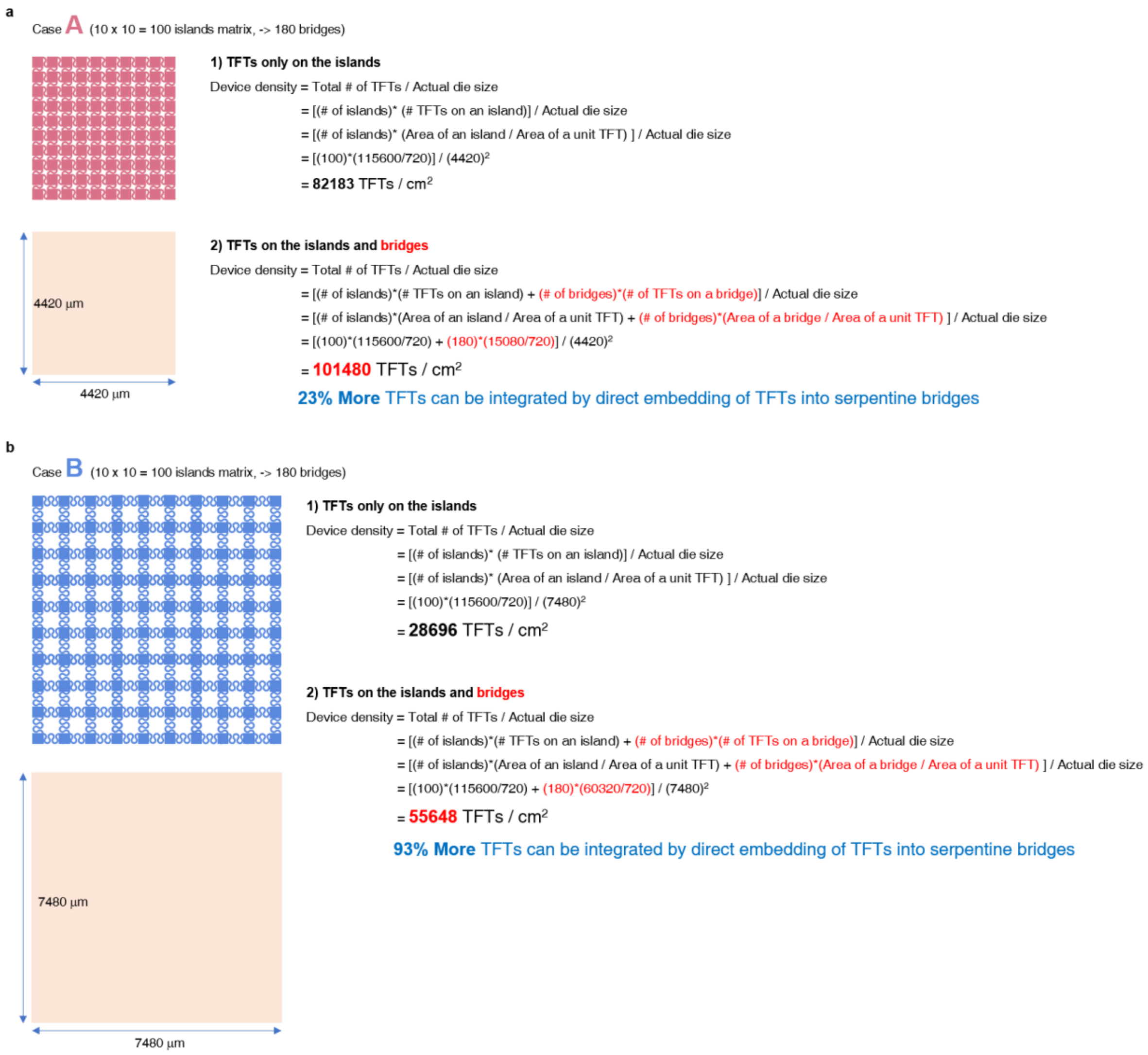

Supplementary Figure 2 | Calculation of integrated density for combination of rigid islands and serpentine bridges. a, Case A (the length ratio between square island and bridge is 3 to 1). 23% more TFTs can be integrated into serpentine bridges. **b**, Case B (the length ratio between square island and bridge is 3 to 4). 93% more TFTs can be integrated by using bridge space without sacrificing stretchability. <u>Refer to supplementary Fig. 3 and 4 for dimensions of square island and serpentine bridge.</u>

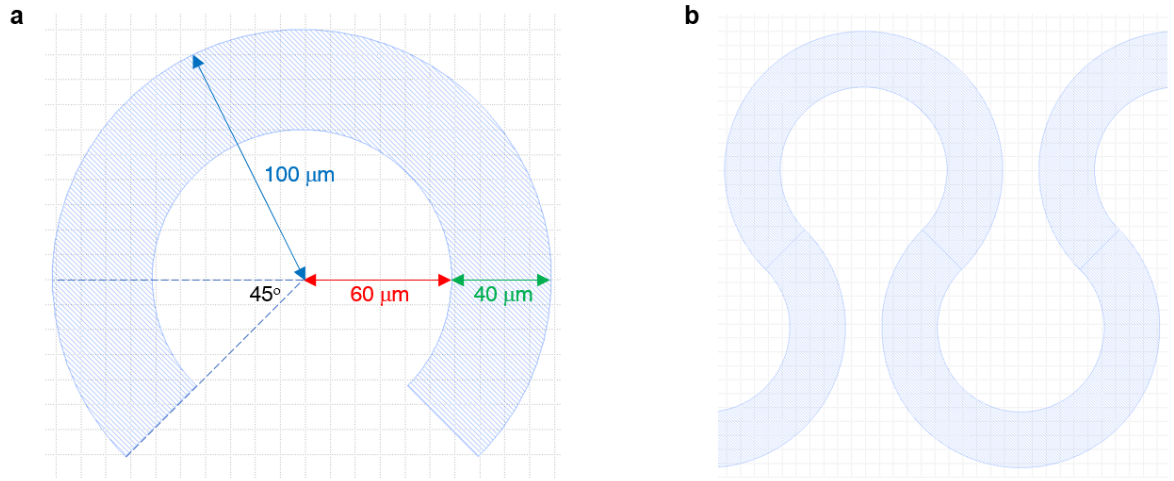

Supplementary Figure 3 | Geometric parameters for serpentine strings. a, A sub-cell for constructing the serpentine bridges. **b**, A string by connecting the sub-cells.

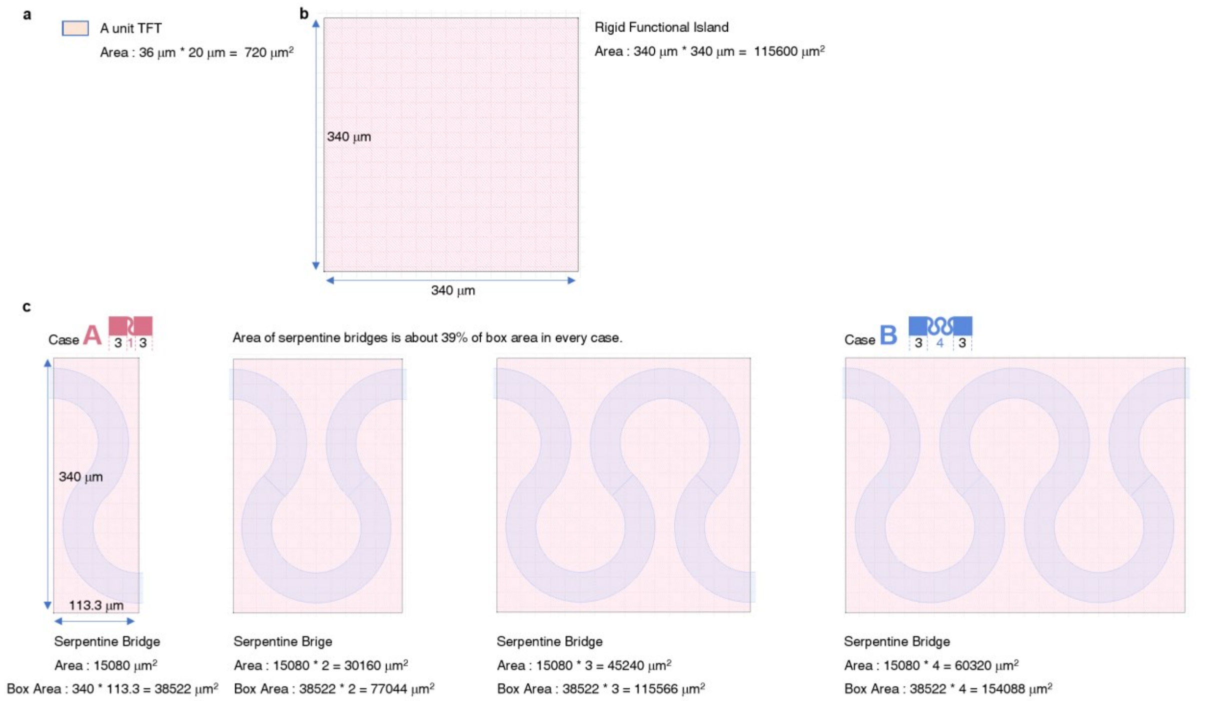

Supplementary Figure 4 | Size comparison among the parts for stretchable electronics. a, A unit TFT. **b**, Rigid functional island. **c**, Serpentine bridges with various lengths (Case A and B). Area of serpentine string is about 39% of corresponding box area.

Ref#	Channel	Mobility	Stretchability	Device density	Year of Publication
30	IGZO	12.83 cm^2/Vs	5 %	204 TFT/cm^2	2013
17	IGZO	11.3 cm^2/Vs	**210 %**	400 TFT/cm^2	2015
31	IGZO	2.07 cm^2/Vs	20 %	Single TR	2015
32	IGZO	0.1 cm^2/Vs	50 %	Single TR	2016
33	IGZO	13.7 cm^2/Vs	5 %	**1092 TFT/cm^2**	2017
19	IGZO	1.2 cm^2/Vs	20 %	36 TFT/cm^2	2018
34	IGZO	1.2 cm^2/Vs	30 %	4 TFT/cm^2	2018
35	IGZO	14 cm^2/Vs	70 %	56 TFT/cm^2	2019
36	IZO	2.24 cm^2/Vs	30 %	58 TFT/cm^2	2019
37	IGZO	Not available, only in changes regarding to unstretched one.	60 %	124 TFT/cm^2	2019
38	IGZO	13.1 cm^2/Vs	30 %	135 TFT/cm^2	2021
39	IGZO	**24.9 cm^2/Vs**	30 %	<1 TFT/cm^2	2022
**-**	**ITO**	**56.2 cm2/Vs**	**100 %**	**30000 TFT/cm2**	**This work**

Supplementary Table 1 | Studies on the stretchable oxide TFTs

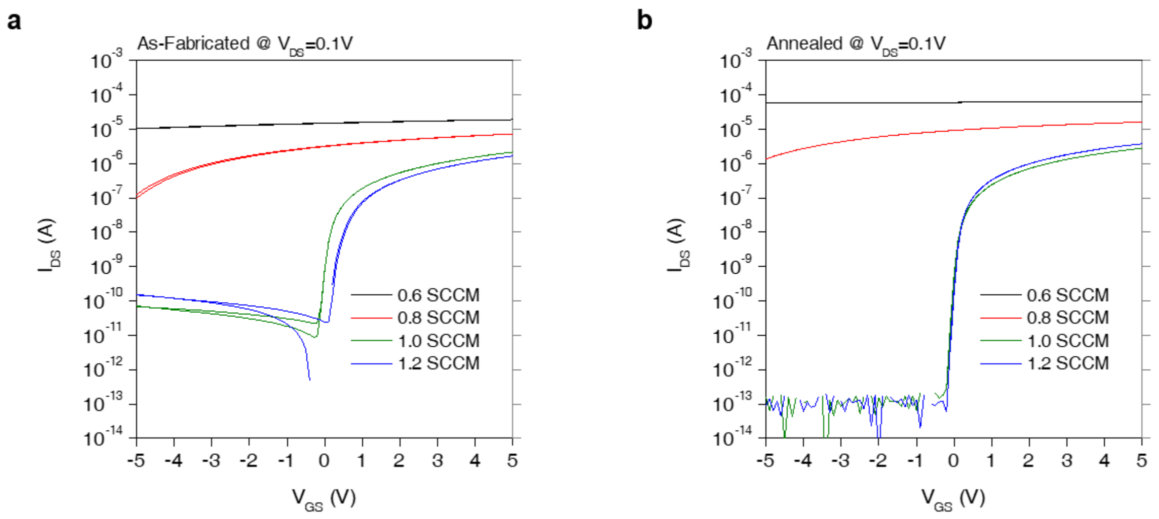

Supplementary Figure 5 | Impact of O_2 flow rate during the deposition of ITO channel layer on the device characteristics. a, Transfer curves measured before thermal annealing. **b**, Transfer characteristics after thermal annealing. With low O_2 flow rates (< 1.0 SCCM), the carrier concentration is too high to be depleted by the gate bias.

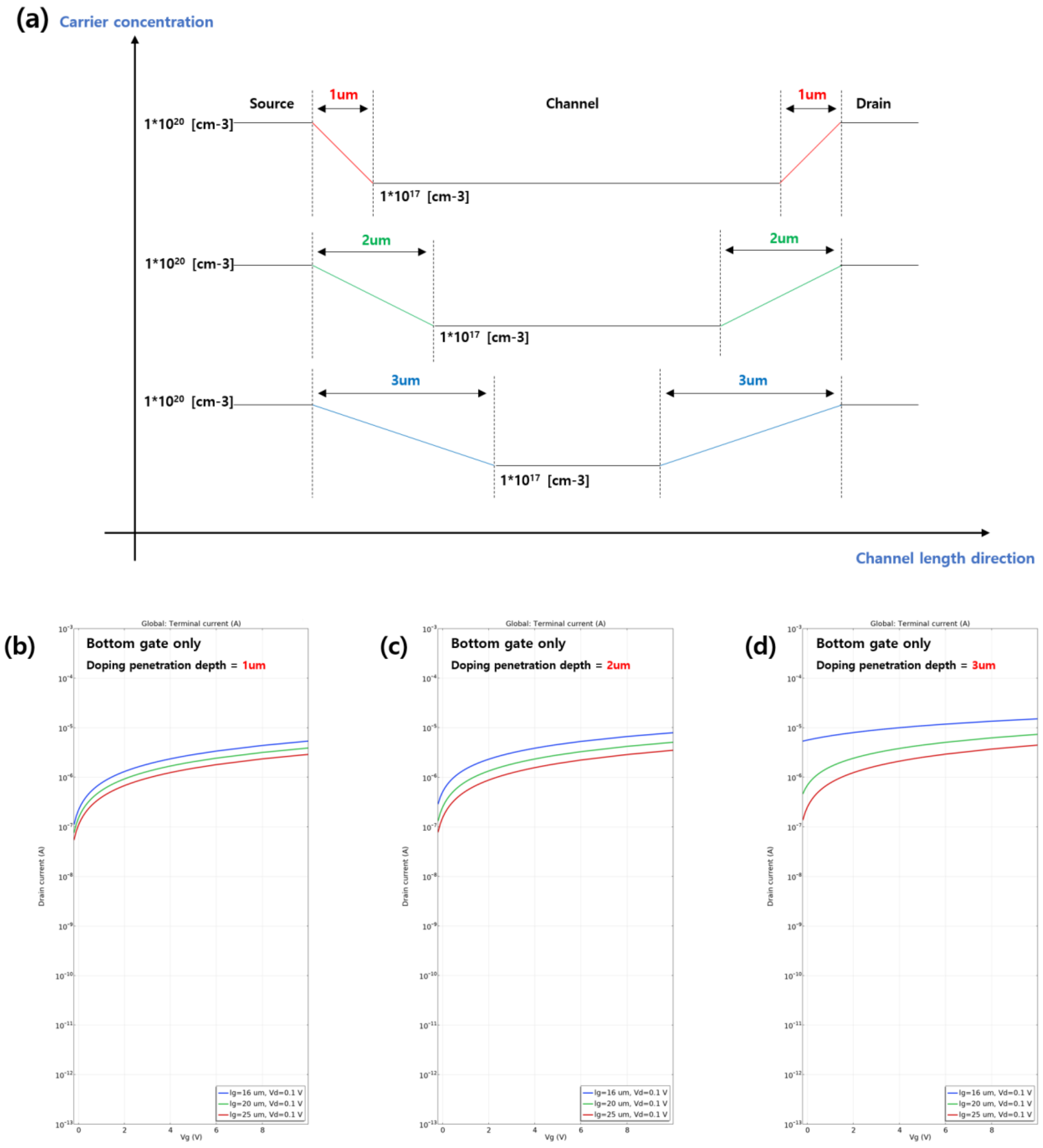

Supplementary Figure 6 | Impact of dopant penetration depth on the TFT characteristics

a, Carrier density profiles with various penetration depths from 1 to 3 μm for device simulation. **b-d**, Simulated transfer characteristics of devices with different penetration depths (from 1 to 3μm).

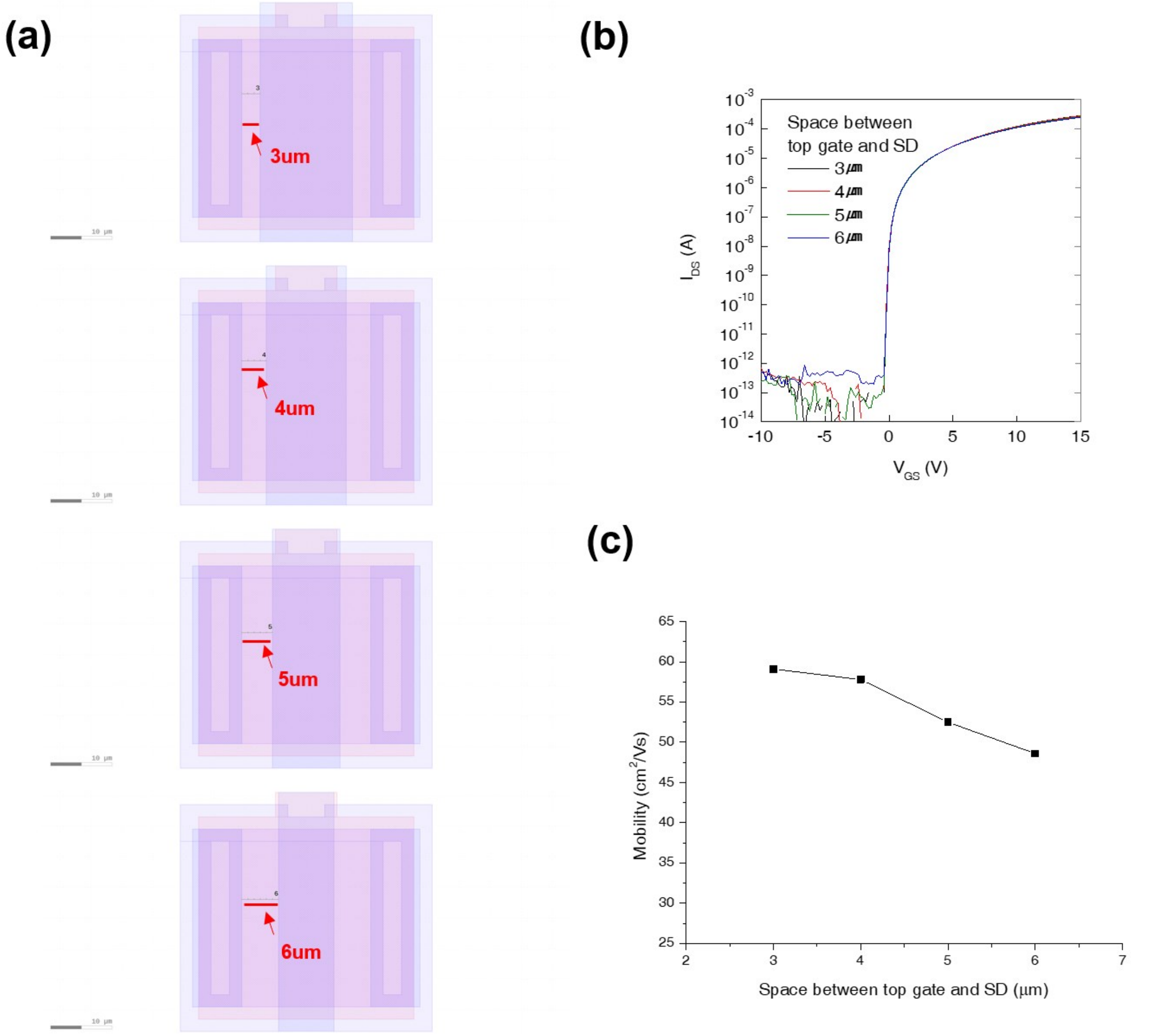

Supplementary Figure 7 | Impact of space between top gate and SD on device characteristics. **a**, Schematics of devices with different widths of gap. **b**, Transfer characteristics of the devices with various gap size. **c**, Field effect mobilities extracted from b.

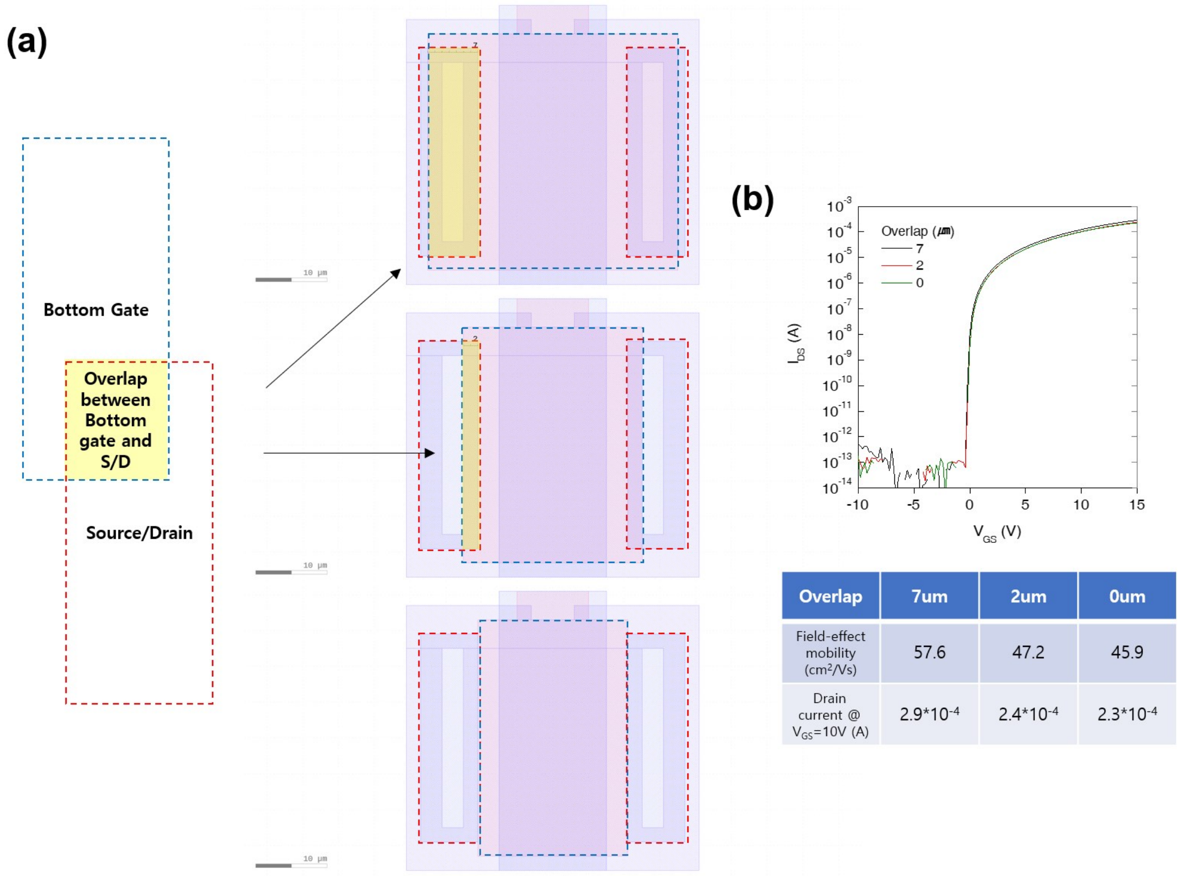

Overlap	7um	2um	0um
Field-effect mobility (cm²/Vs)	57.6	47.2	45.9
Drain current @ V_{GS}=10V (A)	2.9*10^{-4}	2.4*10^{-4}	2.3*10^{-4}

Supplementary Figure 8 | Impact of overlap between bottom gate and SD on the device characteristics. a, Schematics indicating overlapped regions. **b**, Transfer curves and extracted mobility values from them.

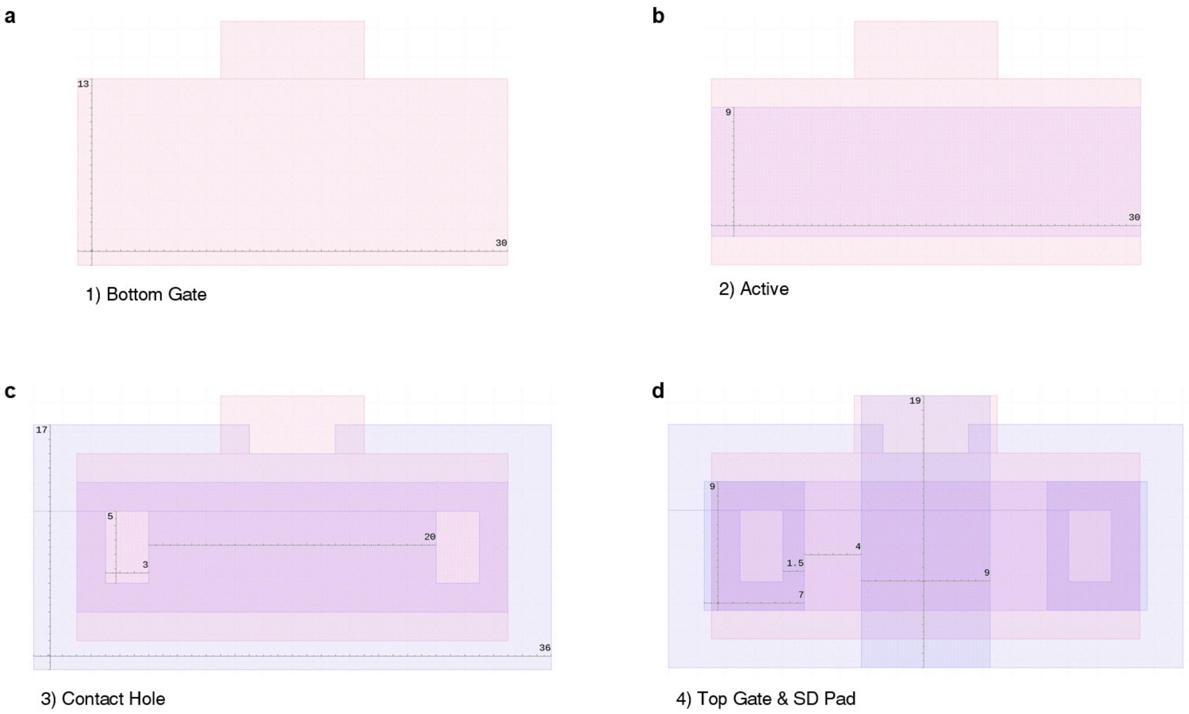

Supplementary Figure 9 | Layout of a unit TFT with dimensions. a, Bottom gate **b**, Active layer **c**, Contact hole. **d** Top gate with SD pad.

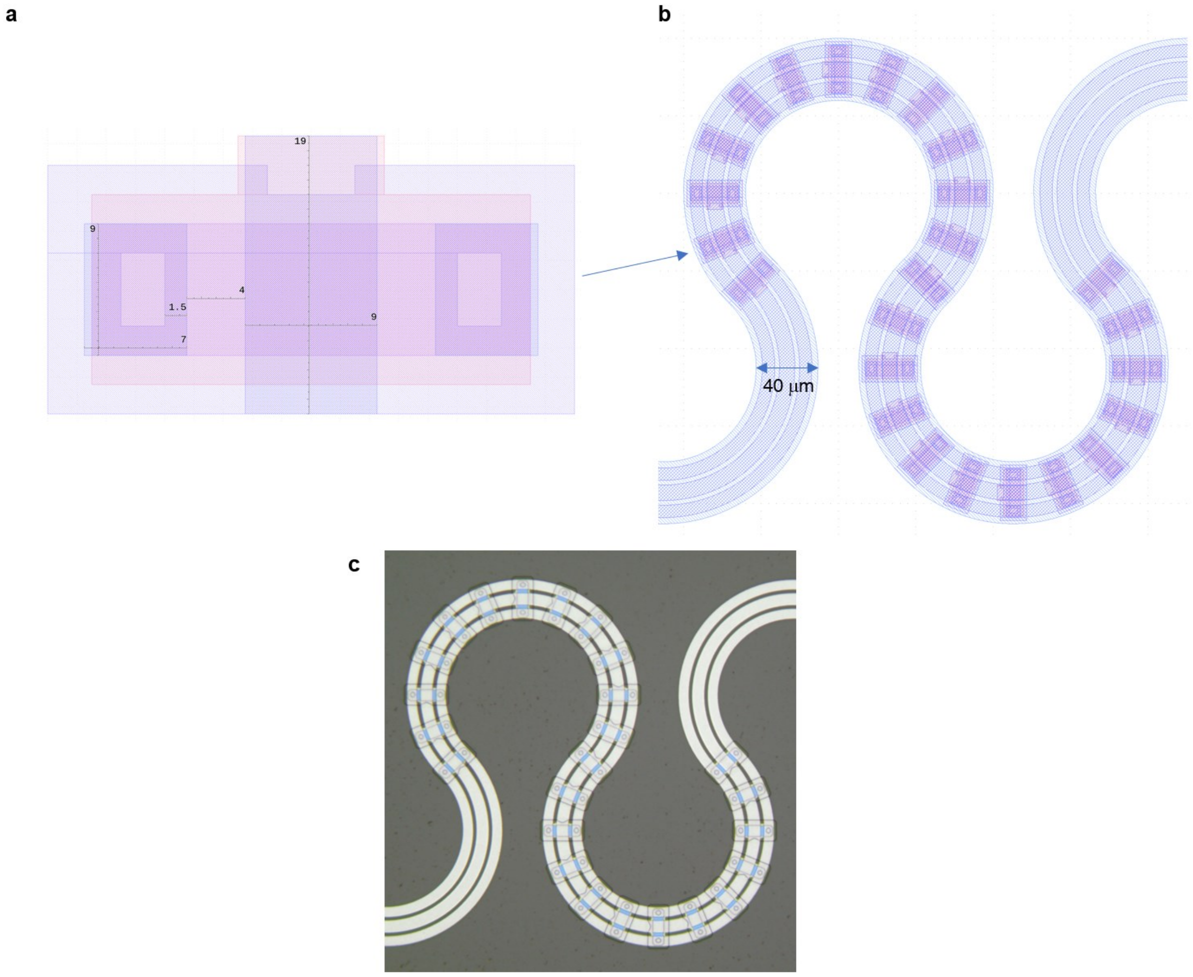

Supplementary Figure 10 | Arrayed 25 unit TFTs on serpentine bridges. a, A unit TFT. **b**, Serpentine string with 25 unit TFTs. **c**, Optical microscopic image of actual TFTs and serpentine metal lines.

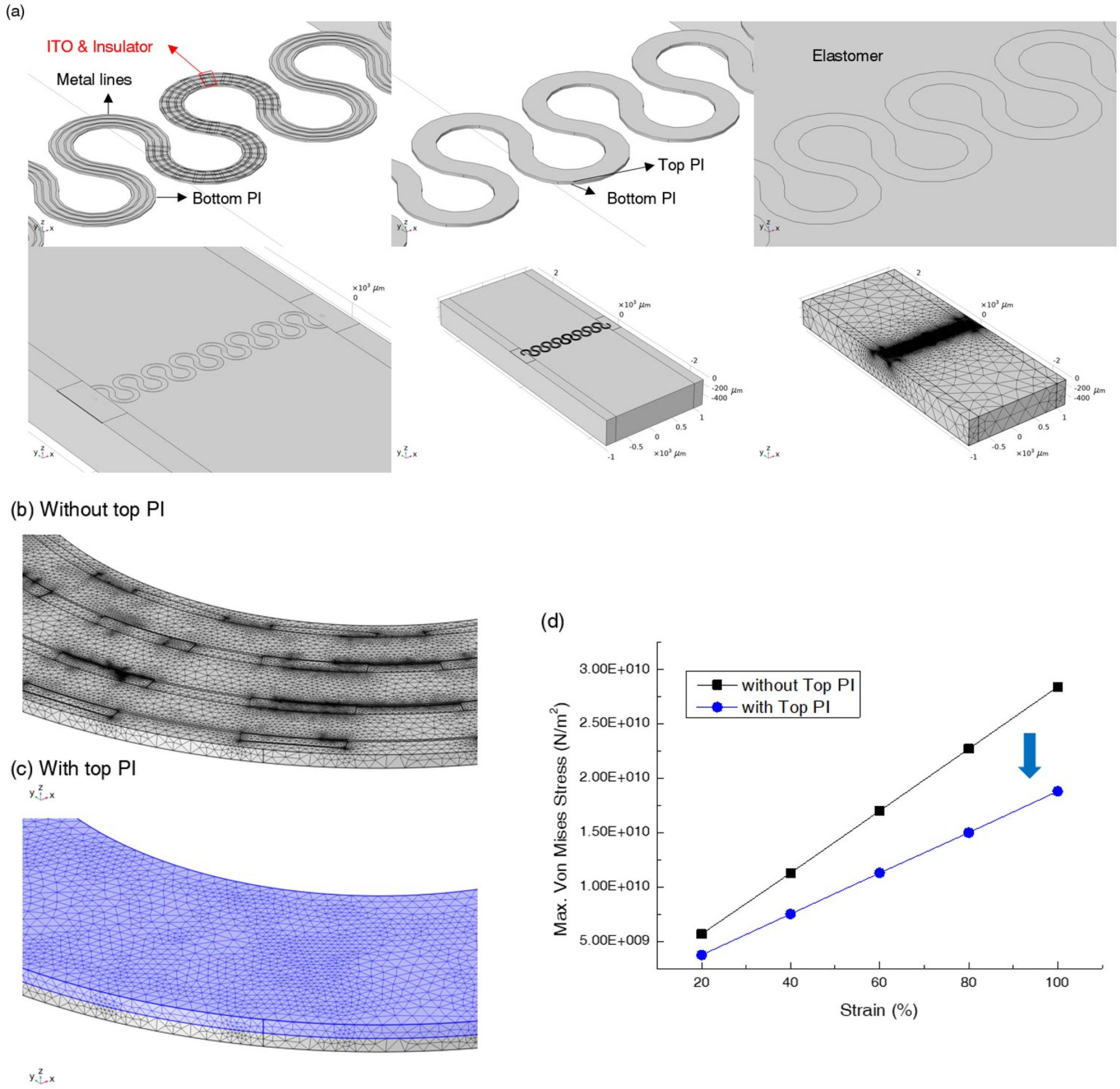

Supplementary Figure 11 | Finite element analyses (FEA) on the deformations of TFT-embedded serpentine string. a, A 3D Model and meshing of it for calculations. **b**, A model of serpentine string without top PI. **c**, A model with top PI. **d**, Maximum Von Mises stress on the stretched devices with and without top PI.

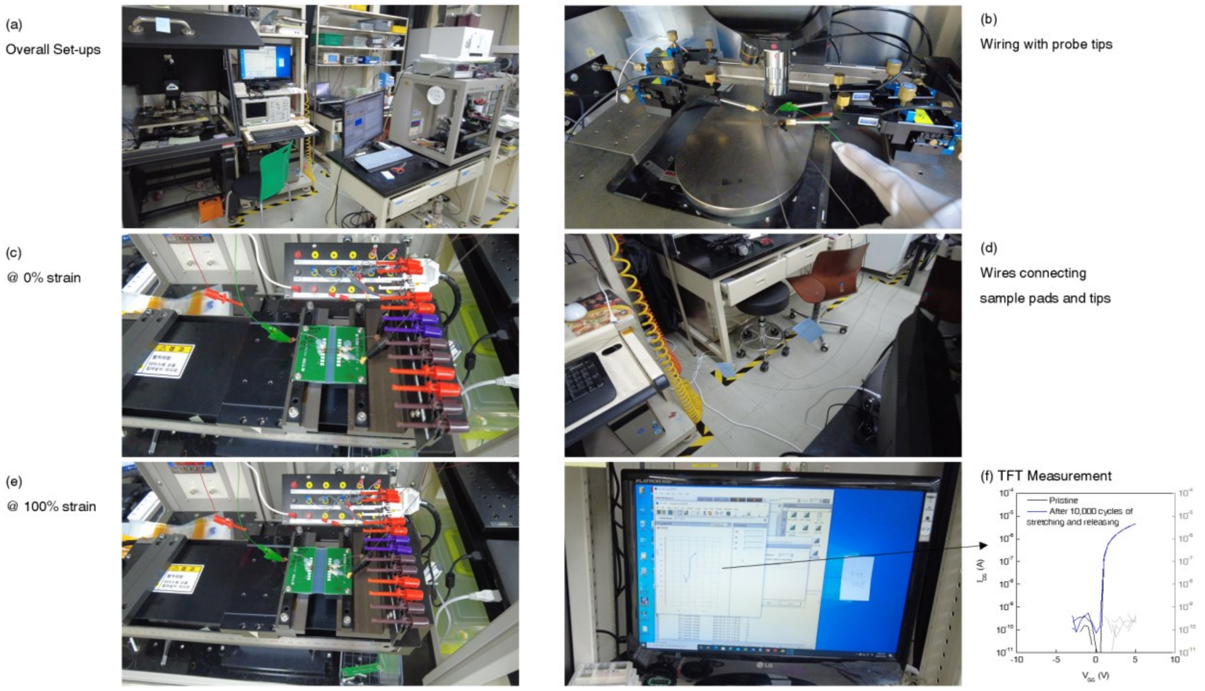

Supplementary Figure 12 | Cyclic stretching test. a, Experimental set-up with sample stage and PC to control the movement of stage. **b**, Wires connected to probe tips in the dark box. **c**, Sample at 0 % strain with wire connected to measurement pads. **d**, Long wires connecting sample pads to probe tips. **e**, Sample at 100 % strain. **f**, real-time measurement of TFT characteristic during the stretching test.

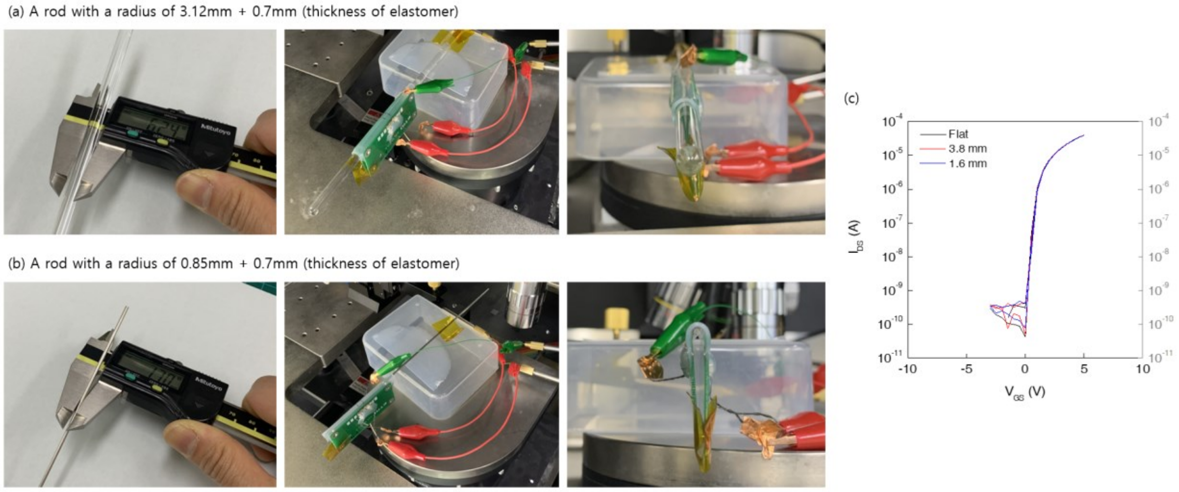

Supplementary Figure 13 | Bending test. a, A rod with a radius of 3.12mm and sample bended along with it. **b**, A rod with a radius of 0.85mm and sample folded along with it. **c**, Transfer curves at different bending radii (Thickness of elastomer (0.7mm) is added to the radius of each rod).

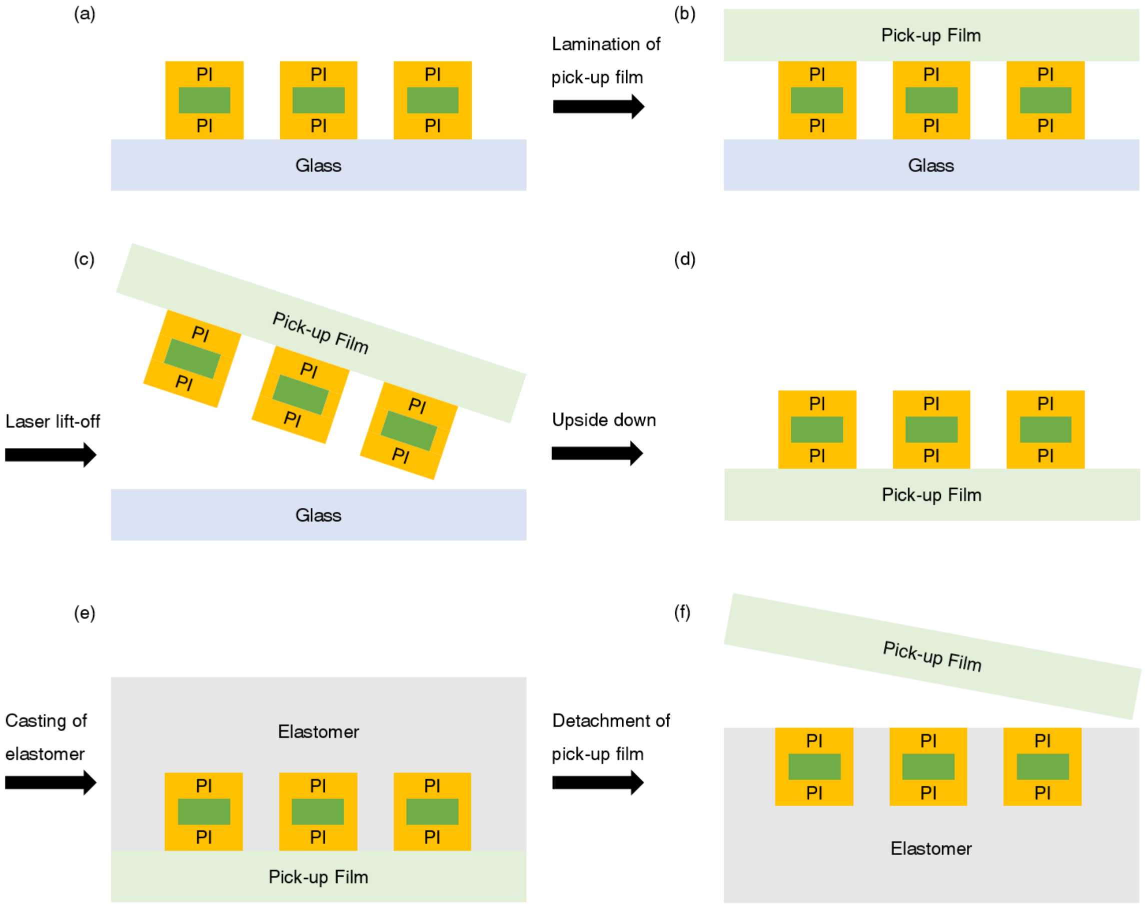

Supplementary Figure 14 | Transfer of fabricated device to elastomer. a, Fabricated devices on the glass wafer. **b,** Lamination of pick-up film on the devise. **c**, Detachment of PI from the glass by laser lift-off technique. **d**, Transferred devices on the pick-up film. **e**, Casting of elastomer on the devices. **f**, Delamination of the pick-up film.
