# Supplementary material for: High density integration of stretchable inorganic thin film transistors with excellent performance and reliability
Source: Nat Commun. 2022 Aug 24;13:4963. doi: 10.1038/s41467-022-32672-8 (PMC9402572; doi:10.1038/s41467-022-32672-8)
Supplement: Supplementary file 2 — Description of Additional Supplementary Files [file 41467_2022_32672_MOESM2_ESM.docx]

**Description of Additional Supplementary Files**

**File Name: Supplementary Movie 1
Description:** LED pixels driven by stretchable oxide TFTs at 0% strain.

**File Name: Supplementary Movie 2
Description:** LED pixels driven by stretchable oxide TFTs at 100% strain.

**File Name: Supplementary Movie 3**

**Description:** Real-time measurement of TFT characteristics during the cyclic stretching test.
